# Subinhibitory concentrations of antibiotics mediate primary metabolism and shape diverse species interactions among coevolved soil *Streptomyces*

**DOI:** 10.1093/ismejo/wrag121

**Published:** 2026-05-23

**Authors:** Molly A Kuhs, Zoe A Hansen, Matthew J Michalska-Smith, Julia R Ahlborn, Linda L Kinkel

**Affiliations:** Department of Ecology, Evolution, and Behavior, University of Minnesota Twin Cities, Saint Paul, MN 55108, United States; Department of Plant Pathology, University of Minnesota Twin Cities, Saint Paul, MN, 55108, United States; Department of Ecology, Evolution, and Behavior, University of Minnesota Twin Cities, Saint Paul, MN 55108, United States; Department of Plant Pathology, University of Minnesota Twin Cities, Saint Paul, MN, 55108, United States; Department of Plant Pathology, University of Minnesota Twin Cities, Saint Paul, MN, 55108, United States; Department of Plant Pathology, University of Minnesota Twin Cities, Saint Paul, MN, 55108, United States

**Keywords:** soil microbiomes, microbial communities, *Streptomyces*, antibiotics, primary metabolism, nutrient competition, subinhibitory concentrations of antibiotics

## Abstract

Antibiotics produced by soil microbes have provided critical advances in human health, yet their roles within natural soil ecosystems remain poorly understood. Though traditionally viewed as inhibitory weapons, antibiotics at subinhibitory concentrations (SICA) can alter transcription across a wide range of gene targets, with documented impacts on microbial metabolism and hypothesized consequences for community resource competition. However, work to date has focused primarily on SICA outside of the context of coevolved microbial populations. As interest in leveraging the power of microbiomes grows, a deeper understanding of the roles of SICA within natural, cooccurring communities is critical to our ability to both predict and harness microbial dynamics. In this work, we explored the impacts of SICA on the primary metabolism of sympatric populations of soil *Streptomyces* sourced from high- and low-nutrient soil habitats. Within each population, the effects of six antibiotic compounds at SICA were quantified on both nutrient-use phenotypes and pairwise resource competition. Overall, isolates from low-nutrient soil were found to be more resilient to changes in primary metabolism in the presence of SICA compared to isolates from high-nutrient soil. Across both populations, SICA significantly modified apparent resource competition among coevolved *Streptomyces* with individual isolates experiencing both increases and decreases in resource use overlap with sympatric partners. The diverse phenotypic shifts observed in this work emphasize that SICA can mediate a broad spectrum of competitive outcomes and highlight the importance of long-term nutrient history in shaping the functional role of antibiotics within microbial communities.

## Introduction

Since their discovery, antibiotics produced by soil-dwelling microbes have been co-opted for drug development and disease suppression to support plant, animal, and human health [1, 2]. However, the role and functions of antibiotic compounds within natural soil communities remain poorly understood. Antibiotics have traditionally been viewed as weapons utilized by soil microbes to inhibit competitors [3]. Yet, recent research has demonstrated that antibiotics significantly alter transcription, metabolism, and microbial phenotypes even at low, subinhibitory concentrations [4–6]. Thus, antibiotics have been defined as having a hormetic functional response: antagonistic weapons at high concentrations and transcription-altering signals at low concentrations [7–9]. Despite examples of transcriptional changes induced by subinhibitory concentrations of antibiotics (SICA), we still know little about the role of SICA in mediating species interactions or the ecological and evolutionary factors shaping their impact within microbial populations [10]. As interest in leveraging the power of microbiomes grows across clinical and agricultural settings, a deeper understanding of the role of SICA in natural communities is critical to predicting microbial dynamics and identifying selective environments most likely to produce novel antibiotic compounds and phenotypes [11].

Recent work has shown that concentrations of antibiotics in natural environments frequently fall well below inhibitory levels (sub-MIC) [12]. This suggests that rather than antagonistic weapons, antibiotic compounds may commonly function as interspecies signaling agents mediating microbial species interactions [13]. As soil bacteria exist as part of complex communities shaped by strong competition for limited resources, SICA have been suggested to play a significant role in shaping primary metabolism, resource use, and competitive dynamics among coevolved populations [1, 3, 14, 15]. Previous work has demonstrated that the presence of SICA can alter individual isolate nutrient use, resulting in reduced niche overlap within microbial populations [16]. These observed shifts in nutrient use highlight the potential for SICA to stimulate neutral or even cooperative interactions that facilitate multi-species coexistence [16, 17]. However, SICA have also been shown to increase negative interactions, giving some individuals a competitive advantage over others [18, 19]. Within the context of a community, it is likely that there is a mixture of positive and negative impacts of SICA on both individual and collective resource use and competition [13, 20, 21]. This spectrum of responsiveness highlights the multifunctional roles of antibiotics in natural systems and suggests a greater complexity beyond the established framework of “signal” or “weapon”. Moreover, although there are documented instances of positive, neutral, and negative impacts of SICA on individual phenotypes, few studies have attempted to contextualize these responses within community nutrient availability and among coexisting populations of microbes. This leaves a critical gap in our understanding of how environmental factors shape microbial dynamics among populations and limits our ability to predict microbial interactions in natural settings [10, 22].

In this work, we sought to quantify the impacts of SICA in shaping primary metabolism among coevolved populations of microbes assuming an isolate’s exposure, production, and metabolic response to antibiotic compounds are intertwined with population history and local habitat [23]. As resource competition is a key driver of species interactions in the soil, we hypothesized that the nutrient context within which a population has evolved may be a critical factor mediating antibiotic roles in natural systems [24, 25]. Nutrient habitat has been shown to strongly influence both primary metabolism (changes in nutrient-use profiles) and secondary metabolism (increases or decreases in inhibitory interactions; [25–29]). Thus, we predicted that responses to SICA on primary metabolism and competitive fitness will vary between microbial communities from soils with distinct nutrient histories.

Here, we evaluated SICA’s impact on nutrient use and niche overlap among coexisting *Streptomyces* to better understand functional roles of SICA within microbial populations. *Streptomyces* (phylum: *Actinobacteria*) are ubiquitous soil bacteria renowned for their capacities to produce diverse antimicrobials and have previously been shown to vary their nutrient-use and antibiotic phenotypes in response to diverse biotic and abiotic factors [16, 27, 30–32]. Utilizing coexisting populations of *Streptomyces* isolates from high-nutrient (amended) and low-nutrient (non-amended) soils, we quantified nutrient-use phenotypes and apparent community competition in the presence and absence of subinhibitory concentrations of six *Streptomyces*-derived antibiotics spanning a range of inhibitor targets. Across 13 000 isolate–antibiotic–nutrient combinations, we quantified the impact of SICA on primary metabolism and nutrient-use dynamics within natural soil populations. Together, these results underscore the importance of integrating community interactions, nutrient habitat, and evolutionary history into our understanding of microbial community dynamics.

## Materials and methods

### Nutrient treatments and bacteria isolation

*Streptomyces* were isolated from a successional grassland prairie community within a long-term nutrient manipulation experiment at Cedar Creek Ecosystem Science Reserve in East-Central Minnesota [33]. In this experiment, nutrient amendments were applied twice annually to 4 × 4 m plots located 1.4 m apart within a 0.3-acre field. Beginning in 1982, both plots received a basal nutrient treatment without nitrogen (P_2_0_5_, K_2_O, CaCO_3_, MgSO_4_, and trace minerals), whereas the nutrient amended (high nutrient) plot received an additional nitrogen amendment (NH_4_NO_3_) at 10 g/m^2^/yr. After 18 years of treatment, soil samples were collected from the center of each plot via a single homogenized soil microcore (1 × 10 cm). Microcores were collected from subsurface soil, beginning 10 cm below the aboveground biomass layer, to significantly limit recent dispersal via soil movement and to sample at a spatial scale consistent with prior evidence of coevolution [27, 34–37]. At the time of collection, soil nitrogen content (NO_3_-N) was significantly greater within the nutrient amended plot, whereas other soil characteristics, including pH, potassium, total carbon, and phosphorus, did not differ significantly between locations (Supplementary Table S1). From the soil core taken at each location, a random collection of *Streptomyces* isolates were cultured, purified, and sequenced to confirm identity (Table S2) [37]. Spore suspensions of each isolate were stored in 20% glycerol at −80°C prior to experimental work. A total of 20 *Streptomyces* with 10 isolates per treatment were randomly selected to be utilized in this study. Alone and in combination with 6 antibiotics, 20 selected isolates resulted in the quantification of 140 unique isolate–antibiotic combinations. Measured across 96 carbon sources, this yields 13 440 quantified phenotypes, expanding the scale of measured SICA data well beyond what has been previously reported.

### 16S rRNA gene sequencing

DNeasy UltraClean Microbial Kit (QIAGEN) was utilized for DNA extraction and 16S rRNA gene sequences were amplified using broad eubacterial primers 27F (5′-AGAGTTTGATCCTGGCTCAG-3′) and 1391R (5′-GACGGGCRGTGWGTRCA-3′) and submitted to the University of Minnesota Genome Sequencing Center (https://genomics.umn.edu) for Sanger Sequencing. Forward and reverse sequences for each isolate were aligned in the Geneious software program (https://geneious.com). Consensus sequences were aligned using MUSCLE [38], and all gaps were removed for a final alignment length of 1117 bp. MEGA X was used to determine the best fit (HKY85) and to generate a maximum-likelihood phylogenetic tree using RAxML with bootstrap support determined using 1000 samples [39]. Taxonomy was assigned to all sequences using NCBI blastn suite against the 16S rRNA sequence database (accessed April 2026; https://blast.ncbi.nlm.nih.gov/Blast.cgi; Table S2). Phylogenetic analysis was performed in R utilizing the “ape” package version 5.7 [40] and “phytools” package version 2.0 [41] (Fig. S1). All consensus sequences have been deposited in GenBank under accession numbers PZ282427–PZ282446.

### Antibiotic treatments

This work utilized six antibiotics commonly found in the local soil and produced by or derived from *Streptomyces* [16]. Antibiotic compounds were selected to represent inhibitors having distinct targets, including 30S ribosomal (streptomycin, tetracycline, and streptothricin), 50S ribosomal (chloramphenicol), cell wall synthesis (vancomycin), and RNA synthesis (rifampicin). Minimum inhibitory concentrations (MICs) were determined for each isolate–antibiotic combination (20 × 6 = 120 combinations) on a solid, nutrient-rich *Streptomyces* medium ISP2 [42] amended with filter-sterilized antibiotic stocks [43] following previously published methods [16]. Briefly, antibiotics were incorporated across a two-fold dilution series into solid medium and four replicate cultures of purified *Streptomyces* spores at 10^8^CFU/ml were dotted on each plate and incubated at 28°C for 72 h. Lowest concentration plates with no visible growth on replicate spots was determined as MIC (Table S3). The SICA was calculated as 10% of the specific isolate–antibiotic MIC, as previously defined in the literature [5, 16]. Values were confirmed by vigorous growth of isolates on ISP2 medium with each antibiotic at SICA compared against growth of each isolate on non-antibiotic amended ISP2 medium.

### Resource utilization

Isolate resource use was determined utilizing methods described previously [16, 44]. Briefly, isolates were grown on Biolog-SF-P2 microplates containing 95 unique carbon sources across 8 carbon classes (Table S4; Biolog, Inc. Hayward, CA). Biolog plates measure the growth of an individual isolate on single carbon sources by comparing optical densities (OD_590_) of each well against a water control. Plates were inoculated following manufacturer’s instructions. Then, 1.5 ml of purified isolate spore suspension in sterilized water at 10^8^ CFU/ml was combined with each specific antibiotic stock and 0.2% carageen for a final 15-ml volume. One hundred microliters of isolate–antibiotic mixture were immediately inoculated into Biolog plate wells. This procedure was repeated for each antibiotic–isolate combination along with a control at the same concentration without added antibiotic. Only one Biolog plate was inoculated for each isolate–antibiotic combination; however, previous work found no significant differentiation with replicate plates following these inoculation methods, suggesting that replication would likely not significantly change our results [16]. All plates were incubated at 28°C for 72 h at which a Bio-Tek Microplate Reader Synergy HT (Agilent Technologies, Santa Clara, CA) was used to measure the absorbance of each well at 590 nm. To standardize the readings from each plate, the OD of the water control well was subtracted from that of all other wells prior to analysis. Negative absorbance values were set to zero, and differenced OD values <0.005 were considered as no growth.

### Nutrient use and growth phenotypes

Resulting resource use data were utilized to calculate nutrient-use phenotypes (utilized carbon sources, niche width, and total growth) for each isolate alone and each isolate–antibiotic combination. Utilized carbon sources were defined as those with OD >0.005. Niche width (NW) was defined as the count of utilized carbon sources and total growth (TG) as the sum of standardized absorbance readings across all utilized carbon sources. Isolate growth was compared on each individual carbon compound in the presence and absence of SICA. We calculated a difference metric representing the change in growth in the presence of SICA, and a distribution of these difference values were used to calculate a standard deviation across all experimental isolates (SD = 0.06 OD). Resulting observations were categorized into four phenotypes as follows: “induction”: no growth in absence of SICA and growth in presence of SICA; “increase”: some growth in absence of SICA and increase in growth >1 SD in presence of SICA; “decrease”: some growth in the absence of SICA and decrease in growth >1 SD in presence of SICA; and “suppression”: some growth in the absence of SICA and no growth in the presence of SICA. Growth occurring on a carbon compound in the presence and the absence of SICA but difference in growth was <1 SD was classified as “No Change”.

### Escape ratio and competition-free growth

The effect of SICA on nutrient competition was evaluated through the calculation of an individual isolate’s escape ratio via evaluation of competition-free growth (CFG) in the presence and absence of each antibiotic at SICA. CFG was calculated as the sum of excess growth across resources for each isolate in a pair of sympatric isolates *i_a_* and *i_b_* with the following equation:


$$ {CFG}_{ab}=\sum_{n=1}^{95}{\mathrm{OD}}_{590}\left({i}_x,{x}_n\right)-\min \left({\mathrm{OD}}_{590}\left({i}_a,{x}_n\right),{\mathrm{OD}}_{590}\Big({i}_b,{x}_n\right)\Big) $$

That is, for any two isolates *i_a_* and *i_b_*, we define excess growth to be either 0 (for the isolate with the lower optical density) or the difference between the two densities (for the isolate with the higher optical density). This represents the amount of a nutrient that a given isolate is able to utilize beyond the maximum utilization of its competitor.

The escape ratio (ER) was defined as the natural log of the ratio of CFG of a pair of sympatric isolates *i_a_* and *i_b_* as defined above in the presence and absence of SICA.


$$ ER=\ln \left(\frac{CFG_{ab}\ \mathrm{with}\ \mathrm{SICA}}{CFG_{ab}\ \mathrm{with}\mathrm{out}\ \mathrm{SICA}}\right) $$

Thus, when ER >0, the presence of SICA results in a reduction of apparent competition (greater CFG for an isolate when competing with another isolate in the presence of a particular SICA). ER were determined for every isolate in the presence and absences of all six antibiotics, in all pairwise combinations within each population (low and high nutrient). As SICA were applied at 10% of each isolate’s individual MIC, absolute antibiotic concentrations varied across isolates; CFG and ER should therefore be interpreted as indicators of apparent resource competition dynamics rather than direct measures of fitness.

### Statistical analyses

Statistical analyses were conducted in RStudio version 2023.12.0+369 using R version 4.2.2. Phylogenetic community composition was compared between populations using permutational multivariate analysis of variance (PERMANOVA, 999 permutations) with homogeneity of dispersion confirmed via betadisper, utilizing “vegan” package 2.6-4 [45]. Comparisons between niche width and total growth across SICA were conducted via one-way ANOVAs with Tukey *post hoc* (*P* value *=* .05). Nutrient-use dissimilarity matrices were calculated using Bray–Curtis on control-standardized OD values. Nutrient-use profiles within each community were contrasted between antibiotics using nonmetric multidimensional scaling (NMDS) and PERMANOVA (999 permutations) implemented in vegan. Isolate responsiveness was compared between nutrient conditions using Welch two-sided *t-*test (*P* value *=* .05). All other comparisons of phenotype prevalence were conducted with Pearson chi-squared test and standardized residuals using “vcd” package 1.4-12 [46]. Pearson chi-squared with simulated *P* values (2000 replicates) was used for pairwise CFG phenotypes due to low occurrence frequencies (Fig. 4b).

## Results

### Subinhibitory concentrations of antibiotics significantly alter nutrient-use phenotypes among *Streptomyces* from high-nutrient, but not low-nutrient, soils

**Figure 1:**
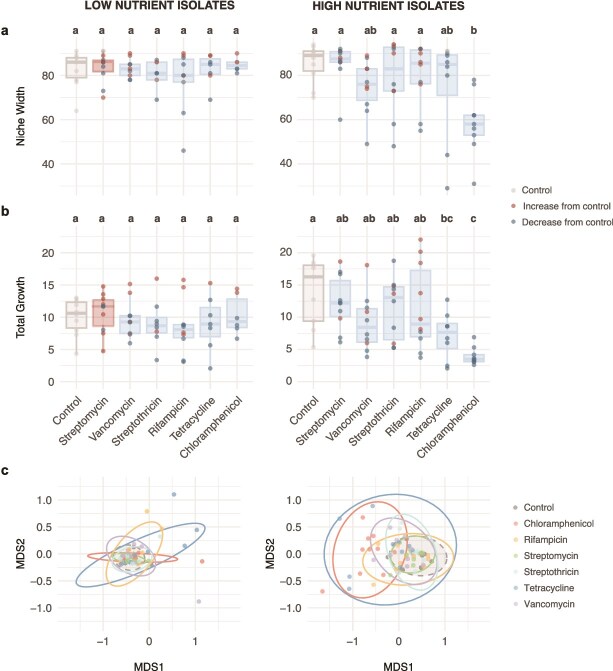
Changes in nutrient-use phenotypes in response to subinhibitory concentrations of antibiotics (SICA) in sympatric *Streptomyces* isolates collected from low- and high-nutrient conditions. **a** Niche width: the count of utilized carbon sources, **b** total growth: the sum of all standardized absorbance readings across all utilized carbon sources, and **c** nonmetric multidimensional scaling (NMDS) ordination variation in isolate nutrient use (*P* value = .08 low nutrient, *P* value = .001 high nutrient). Data represent ordination based on Bray–Curtis dissimilarity among 70 isolate–SICA combinations in each panel. For all panels: gray coloring represents phenotypes in the absence of SICA (control), points represent response of individual isolates (*n* = 10 per bar/NMDS group), and letters denote statistical significance (*P* value < .05). Isolates with low overall growth rate (<2 summed OD_590_) are not displayed.

Overall, isolates from low-nutrient soils (LNI) were less sensitive than isolates from high-nutrient soils (HNI) to inhibition by the six antibiotics tested (Table S3, two-way ANOVA, *P* value = .09). LNI had mean MICs averaging 50% higher than HNI though median MIC values were more similar between the two isolate communities (Table S3). To quantify changes in primary metabolism, isolate nutrient-use phenotypes (niche width and total growth) were evaluated across 95 unique carbon sources in the presence and absence of six SICA. Both HNI and LNI frequently had reductions in average niche width and total growth in the presence of SICA. However, only HNI saw statistically significant reductions in niche width or total growth, though the extent of the shifts in phenotype varied by antibiotic (Fig. 1a and b, one-way ANOVA, *P* value_NW-L_ = .58, *P* value_NW-H_ = .003, *P* value_TG-L_ = .81, *P* value_TG-H_ < .001). Compared to control, HNI experienced significant reductions in average niche width in response to chloramphenicol ($\Delta{\overline{x}}_{\mathrm{nw}}=-28\%$, *P* value = .002) and in total growth from both tetracycline ($\Delta{\overline{x}}_{\mathrm{tg}}=-48\%$, *P* value = .017) and chloramphenicol ($\Delta{\overline{x}}_{\mathrm{tg}}=-68\%$, *P* value < .001). In contrast, many individual isolates from both high- and low-nutrient habitats displayed increases in niche width and/or total growth in response to SICA.

Across all isolate–SICA combinations from both populations, 32% of combinations increased total growth and 33% of combinations increased niche width from control, indicating that isolates often produced greater biomass and/or exhibited growth on specific nutrient sources in the presence of an antibiotic (Fig. 1a and b). Additionally, change in nutrient-use profiles in the presence versus absence of SICA was characterized via NMDS ordination of isolate resource use (Fig. 1c). SICA resulted in an increased differentiation in nutrient use from control for HNI, but not LNI (Fig. 1c, *F* = 3.5 and *P* value = .001, *F* = 1.4 and *P* value = .08, respectively). Specifically, among HNI, the presence of tetracycline and chloramphenicol drove the greatest differentiation in nutrient use profiles from control, suggesting that SICA play functionally distinct roles and vary by community. With 16S rRNA gene sequencing, HNI and LNI isolates were broadly intermixed across the phylogeny with no clear separation by soil origin (Fig. S1). Community membership did not significantly predict phylogenetic composition (PERMANOVA *F* = 2.5, *R*^2^ = 0.12, *P* value = .094), indicating that the differential responses to SICA between communities are not a straightforward function of underlaying taxonomic differences [35].

### SICA most frequently result in negative growth responses among *Streptomyces* from high-nutrient soils, whereas *Streptomyces* from low-nutrient soils balance positive and negative growth responses

**Figure 2:**
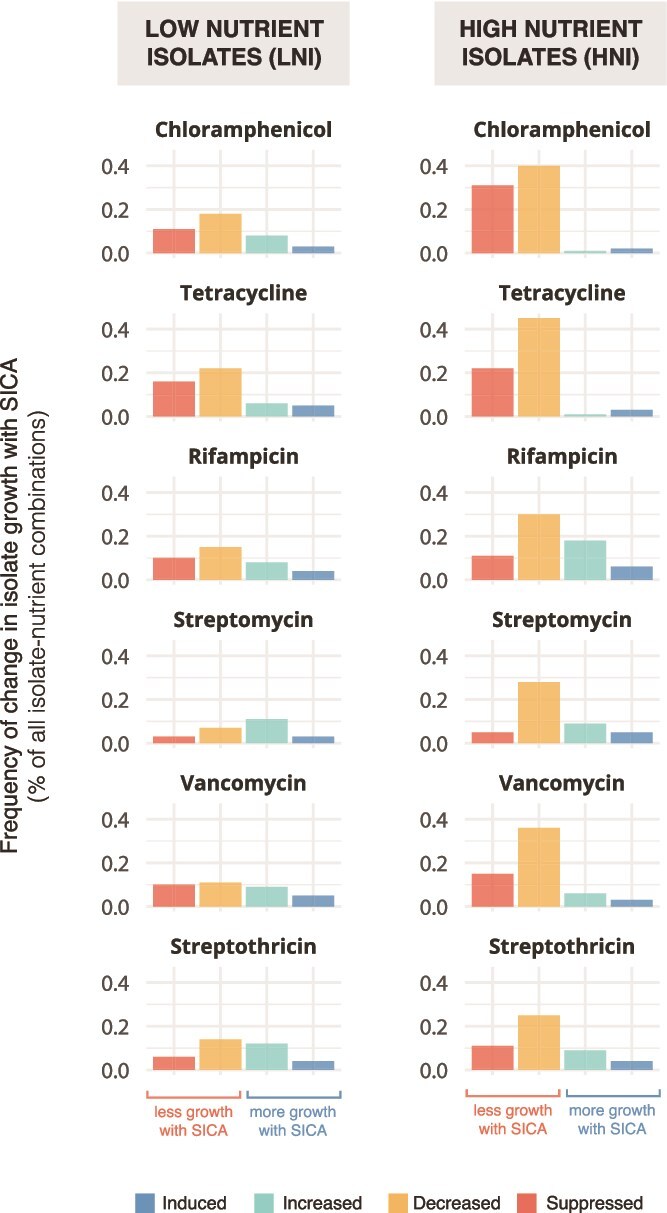
Frequency of changes in isolate growth from control when in the presence of subinhibitory concentration of antibiotic across all isolate–carbon source combinations among both low- and high-nutrient isolates (*n* = 950 per panel). Colors denote categorial phenotypes in the presence of SICA with “induced” defined as no growth in control and growth with SICA, “increase”: greater growth with SICA than control, “decrease”: less growth with SICA than control, and “suppressed” as growth in control with no growth with SICA. Growth difference values <1 SD of overall change in growth (<|0.06| from the control) were classified as “no significant change” and are not shown.

To better understand how SICA alter *Streptomyces* nutrient-use phenotypes, we quantified the frequency and direction of change in isolate growth in response to SICA across all 95 carbon sources (Fig. 2, Figs. S2 and S3). Changes in growth were categorized as “induction” if isolate growth occurred with SICA but not in control (absence of SICA), “suppressed” if growth occurred in control but not in the presence of SICA, and “increase” or “decreased” if isolates grew in both conditions but experienced a statistically significant difference in growth (ΔOD_590_ > ±1 SD) between control and SICA conditions.

Consistent with overall total growth and niche width phenotypes, HNI were more responsive to SICA than LNI across all isolate–carbon combinations (Welch two-sample *T*-test, *P* value < .0001). Among HNI, 60.6% of the total isolate–carbon source combinations resulted in significantly different growth from control in response to SICA, whereas only 36.1% of LNI differed from control with SICA (Figs. S2 and S3). Overall, when categorized by direction of change, negative growth responses to SICA (decrease or suppression in growth) were more common than positive responses (increase or induction of growth) regardless of isolate nutrient history (Fig. 2). Among all SICA, chloramphenicol and tetracycline, the two bacteriostatic compounds tested, resulted in the greatest frequency of negative growth among both HNI and LNI which may suggest that at low concentrations, compounds that impede isolate growth and reproduction have the greatest impact on nutrient use. Overall, negative responses were more frequent among HNI than LNI (71% vs. 29% of carbon sources with chloramphenicol, 70% vs. 38% with tetracycline). Among LNI, positive and negative growth responses occurred at relatively equal frequencies across SICA (Pearson’s chi-squared, *χ*^2^ = 0.30, *P* value = .998) in contrast to the dominant negative growth response among HNI (Pearson’s chi-squared, *χ*^2^ = 15.3, *P* value = .009). Thus, though aggregate nutrient-use phenotypes among LNI did not show significant differences from control (Fig. 1a, b), SICA still influenced primary metabolism and nutrient use among LNI.

### Responses to SICA can both enhance and reduce competition-free growth among sympatric isolates

**Figure 3:**
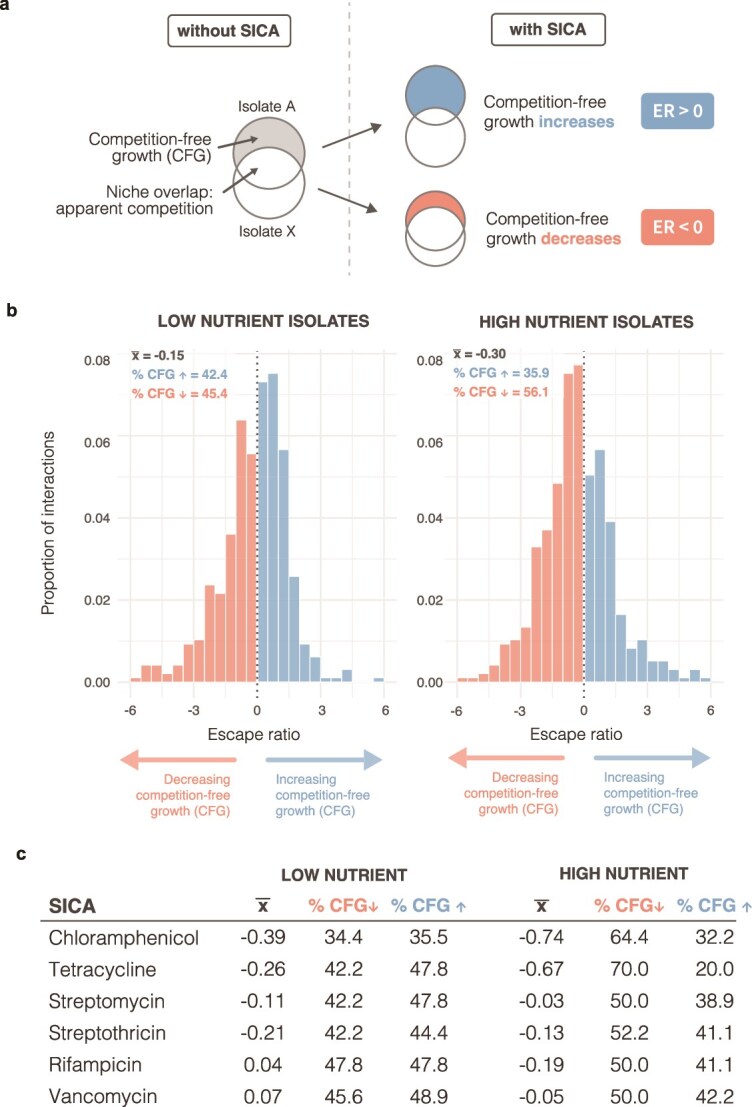
Characterization of changes in competition-free growth among paired isolates in the presence of SICA. **a** Competition-free growth of an isolate A is denoted by a shaded circle, which represents the cumulative growth of isolate A on nutrients the competing isolate X does not use. The escape ratio (ER) is the natural log of the ratio of competition-free growth in the presence vs. the absence of SICA. Thus, an ER >0 denotes an increase in competition-free growth with SICA whereas ER <0 denotes a decrease in competition-free growth with SICA. **b** Proportion of escape ratio values across all possible sympatric isolate combinations among both high- and low-nutrient isolates (*n* = 540/panel). **c** Proportion of escape ratio values split by individual antibiotic (*n* = 90/SICA). For panels B and C, isolate combinations with <|10%| change in competition-free growth were considered no change.

To explore how shifts in isolate growth may impact nutrient competition within a coevolved community, we first quantified an individual isolate’s nutrient-use overlap to all possible sympatric pairwise isolates in the presence and absence of each SICA. Shifts in pairwise nutrient-use overlap were utilized to quantify positive or negative changes in an individual isolate’s competition-free growth (CFG), or escape from apparent resource competition, in response to each SICA (Fig. 3a). Across both HNI and LNI, individual isolate responses to SICA were observed to both increase an isolate’s CFG (increased escape from apparent resource competition) and decrease an isolate’s CFG (decreased escape from apparent resource competition) across all possible sympatric isolate pairs (Fig. 3b, additionally Figs. S4–S6). Specifically, when averaged across all antibiotics, HNI saw a significantly greater proportion of individual isolate responses resulting in reduced CFG (56.1%) compared with LNI (45.4%, Pearson’s chi-squared *χ*^2^ = 21.07, *P* value < .001). Responses to specific antibiotics were not consistent across both populations. For example, tetracycline reduced CFG for 70% of isolates among HNI but in only 42% of isolates among LNI, reinforcing the idea that SICA play variable roles in different communities (Fig. 3c).

Across all antibiotics, responses among LNI consistently resulted in relatively equal proportions of increases and decreases in CFG, whereas HNI responses were more frequently decreases in CFG (Fig. 3c). This contrast in the frequency of decreasing CFG among isolate nutrient histories is particularly notable in responses to chloramphenicol (64.4% vs. 34.4%, Pearson’s chi-squared *χ*^2^ = 3.92, *P* value = .048) and tetracycline (70.0% vs. 42.2%, *χ*^2^ = 15.15, *P* value < .001). Overall, an individual isolate’s response to SICA had a higher frequency of resulting in negative impacts on CFG, particularly among HNI, suggesting that SICA may increase nutrient competition dynamics among isolates evolved in high-nutrient environments.

### Among pairwise interactions, the majority of shifts in nutrient use in response to SICA result in a competitive advantage for at least one isolate

**Figure 4:**
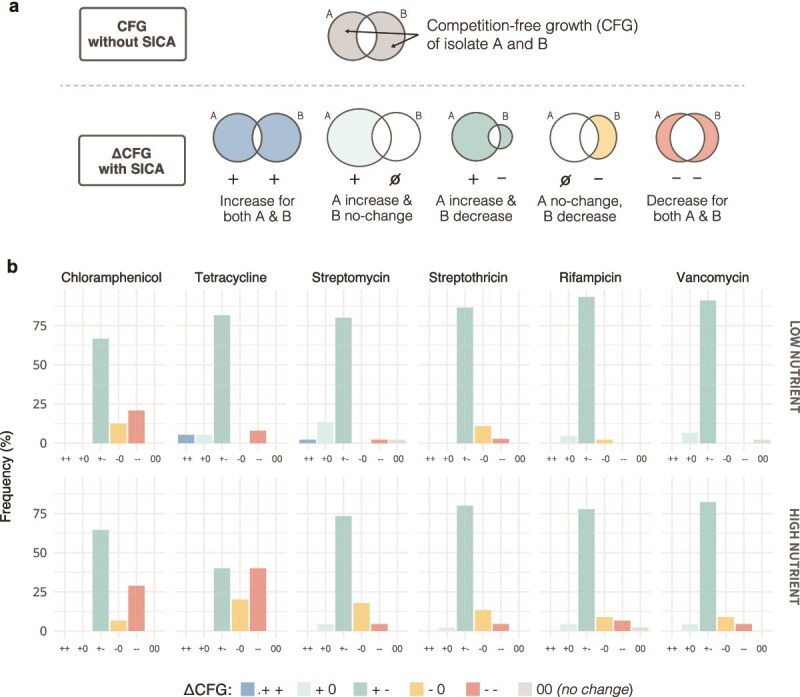
**a** Pairwise shifts in competition-free growth between two isolates, A and B, in both presence and absence of SICA. Each isolate’s niche width is represented by a circle with competition-free growth (CFG) denoted by shaded regions. Symbols and color variants display possible shifts in CFG in response to SICA resulting in five unique phenotypes representing different outcomes in apparent competition (no change for both isolates not shown). Venn diagrams are conceptual examples and do not represent all possible shifts that could produce each phenotype. **b** Frequency of pairwise apparent competition phenotypes across all sympatric isolates in high-and low-nutrient populations (*n* = 90/panel). For each isolate in a pairwise interaction, escape ratio (ER) < |0.1| is defined as “no change” (0) in competition-free growth (CFG) in the presence vs. absence of SICA. ER > 0.1 denotes an increase (+) in CFG with SICA, whereas ER <−0.1 denotes decrease (−) in CFG with SICA.

Within the broader context of resource competition, an individual’s shift in nutrient use in response to SICA may result in a corresponding potential competitive benefit or disadvantage among interacting community members. Here, we define an apparent competitive benefit as an increase in CFG against a pairwise community member in response to SICA and a competitive disadvantage as a decrease in CFG. SICA-induced shifts in CFG were categorically scored as positive, neutral, or negatively impacting apparent competition for both isolates within each pairwise sympatric interaction (Fig. 4a). These pairwise scores were then used to quantify the frequency of shifts in apparent competition within each isolate community and in response to each SICA. The frequency of categorical shifts in CFG significantly differed between HNI and LNI pairs largely driven by the differences in negative interactions (-0 and --) between the communities (Fig. 4b, Pearson’s chi-squared, χ^2^ = 37.1, *P* value < .001). Across both HNI and LNI, a majority of pairwise isolate responses to SICA resulted in an increase in CFG for one isolate and a decrease in CFG for the other (84.6% of isolate pairs among LNI, 69.6% among HNI). There were very few interactions that resulted in mutually (++) or one-sided (+0) positive impact on CFG in response to any of the six antibiotics. Although interactions among LNI resulted in a low proportion of mutually positive interactions (5.3% with tetracycline, 2.2% with streptomycin), we found no instances of mutually positive interactions among HNI for any antibiotic tested. The majority of pairwise interactions resulted in at least one isolate at a competitive disadvantage (+-, 0-, and --) compared to growth in the absence of SICA (92.3% LNI isolate pairs, 84.4% HNI isolate pairs). As the most prevalent phenotype found in both communities results in a competitive advantage for one isolate with a disadvantage for its pairwise competitor (+,-), these data suggest that antibiotics may be significant to mediating resource competitive interactions even at subinhibitory concentrations.

## Discussion

This study characterizes the impacts of SICA on nutrient use and resource competition among coexisting *Streptomyces* isolates to better understand the functional roles of SICA within soil communities from varied resource environments. Overall, SICA induced widespread shifts in isolate growth and nutrient use, highlighting the role antibiotics can play in shaping primary metabolism and competitive species interactions among microbial populations in soil. We found that isolates not only shifted their growth on specific nutrients in response to SICA, but many isolates also demonstrated growth on certain nutrients *only* when in the presence of an antibiotic. This underscores the exquisite sensitivity and flexibility of individual microbes to respond to the environment and highlights the potential for dynamic responses to SICA to mediate species interactions and microbial competition in unexpected ways.

We found that the most significant decreases in nutrient use among individual isolates were in response to subinhibitory concentrations of tetracycline and chloramphenicol. Strong impacts of these two compounds, both ribosomal-targeting, replicate findings in a similar system and strongly suggest that some antibiotic compounds may have greater impact at subinhibitory concentrations than compounds with other inhibitor targets [16]. Our findings could suggest that antibiotic compounds that impair protein function result in metabolic costs such as slower translation or uptake that more directly impact isolate nutrient use than compounds with other targets [47]. Together, these findings provide strong motivation for future research focused a larger-scale evaluation across a range of antibiotic compound types and their impacts for microbial community structures and dynamics.

We found isolate responsiveness to SICA was shaped by the community’s long-term selective habitat. Overall, isolates from low-nutrient soils (LNI) were more resilient to inhibition by the six antibiotic compounds tested and exhibited fewer significant differences in the frequency and magnitude of primary metabolic responses to SICA compared to isolate from high-nutrient soils (HNI). Across both HNI and LNI, the largest reductions in growth in the presence of SICA occurred on N-sourced carbon compounds (Fig. S7). However, these reductions were much greater among HNI, suggesting that the long-term history of nitrogen amendments influenced individual isolate responses to SICA and highlight the importance of nutrient availability in shaping both the long-term evolution of microbial communities and short-term ecological responses [48]. In previous work, isolates taken from nutrient-rich environments were associated with smaller niche widths and greater nutrient use specialization [27]. Our data have expanded this finding, showing that the realized niches in the presence of SICA among HNI were significantly lower than among LNI (Fig. S8). Thus, over evolutionary time, isolates from high-nutrient spaces may have stronger constraints on nutrient utilization, limiting their ability to alter nutrient use in the presence of SICA. However, isolate responsiveness to SICA may not be limited to changes in primary metabolism, and reductions in niche widths among HNI may be correlated with indirect effects on nutrient use such as the metabolic costs of resistance or the production of antibiotics and other secondary metabolites [17, 19, 44, 49, 50]. Future work exploring responsiveness of secondary metabolism to SICA considering carbon context and nutrient diversity important covariates will be important in disentangling the role of SICA across ecological and evolutionary time scales.

Although SICA are recognized to influence phenotypes of individual microbes, there is little understanding of the breadth and depth of phenotypic responses and how they mediate species interactions in complex, multi-species communities [30, 31, 51]. Previous work has suggested the potential for SICA to mediate niche differentiation and optimize overall community nutrient use toward multi-species coexistence [16]. In this scenario, shifts in niche width response to SICA could be expected to be beneficial or neutral to coexisting populations, with isolates adjusting primary metabolism and nutrient use in response to community members in ways that may reduce competitive conflicts. However, in this work, we found very few interactions that resulted in mutually positive impacts on apparent nutrient competition among isolates. Rather, the majority of isolate pairs from both HNI and LNI resulted in a competitive detriment for one if not both isolates, suggesting that rather than supporting coexistence, antibiotics at subinhibitory concentrations appear to be functioning as competitive weapons targeting primary metabolic interactions. Thus, even if they are not being used in direct antagonistic inhibition, antibiotics still may function as weapons shaping competitive species interactions [52].

Historically, the ecological roles of antibiotic compounds in natural systems have been characterized by hormesis or capable of eliciting a differential phenotype at high versus low concentrations [5, 7, 8]. Antibiotics at high concentrations can act as antagonistic weapons that kill or impede the function of other microbes, whereas antibiotics at low concentrations can induce transcriptional changes that may be positive or negative to impacted populations [4, 8, 13, 53]. However, rather than the dichotomy between weapon and signal, dynamic shifts and responses to various concentrations of antibiotics allow for increasingly complex interactions shaped by both biotic and abiotic factors. Our work shows that among coexisting isolate pairs, responses to SICA most frequently resulted in a competitive benefit to one isolate and a loss to the other. Although quantification of these pairwise comparisons may suggest SICA being utilized as weapons, individual isolates frequently saw both positive and negative outcomes across all measured interactions. That is, isolates that received a competitive advantage with SICA in some sympatric pairings resulted in a competitive loss with the same SICA in other pairings, suggesting that responses are likely shaped by a multitude of interactions among coexisting community members [54–56]. Further, microbes in natural settings are part of interconnected communities in which spatial and temporal dynamics will influence both the range of antibiotic concentrations and individual exposures [23, 57]. Modification of one behavior or phenotype will lead to cascading shifts and effects across multiple interconnected members and likely influence competitive dynamics, community structure, and overall evolutionary trajectories of polymicrobial communities well beyond two-way species pairs [56]. Our findings emphasize that further study of SICA in mediating interspecies interactions—together with the characterization of a broad spectrum of interactions beyond the signal versus weapon dichotomy—is critical for deepening understanding of the complex dynamics and structure of polymicrobial communities [56].

In summary, we found that SICA significantly altered primary metabolism and apparent resource competition among *Streptomyces*, though in idiosyncratic ways. Long-term soil nutrient habitat played an important role in shaping individual isolate responses to SICA, with isolates coevolved under low-nutrient soils having greater primary metabolic resilience (fewer change in primary metabolic phenotypes in response to SICA) compared to isolates from high-nutrient soils. The diverse phenotypic shifts in response to SICA observed throughout this work emphasize that antibiotics can mediate primary metabolic interactions in complex ways among multiple coexisting community members simultaneously. Overall, our findings suggest antibiotics are not easily fit into monofunctional roles of signal or weapon but rather play highly dynamic roles in shaping and mediating species interactions across complex nutrient landscapes. Our study shows the dynamic functional roles of antibiotics within both the evolutionary history and current ecological context of interacting populations, and greatly supports future work aimed at improving predictions of complex microbial dynamics relevant to plant, animal, and human health.

## Supplementary Material

KuhsSICA_supplement_final_wrag121

## Data Availability

16S rRNA gene sequences for all isolates have been deposited in GenBank under accession numbers PZ282427–PZ282446. All datasets and analytical code underlying this study are publicly available on GitHub (https://github.com/makuhs/SICA).
