# Supplementary material for: Subinhibitory concentrations of antibiotics mediate primary metabolism and shape diverse species interactions among coevolved soil Streptomyces
Source: ISME J. 2026 May 23;20(1):wrag121. doi: 10.1093/ismejo/wrag121 (PMC13285870; doi:10.1093/ismejo/wrag121)

Supplementary martials for:

Subinhibitory concentrations of antibiotics mediate primary metabolism and shape diverse species interactions among coevolved soil *Streptomyces*

Molly A. Kuhs[1*], Zoe A. Hansen[2], Matthew J. Michalska-Smith[1 2], Julia R. Ahlborn[2], Linda L. Kinkel[2]

1. Department of Ecology, Evolution and Behavior, University of Minnesota, Saint Paul, MN
2. Department of Plant Pathology, University of Minnesota, Saint Paul, MN

[*]**Author for Correspondence:** Molly A. Kuhs
Address: Department of Ecology, Evolution and Behavior, University of Minnesota, 1479 Gortner Ave, Saint Paul, MN 55108; Email: kuhsx003@umn.edu

Table S1: Soil characteristic data taken at time of sampling from soils which *Streptomyces* were isolated. High nutrient isolates (HNI) utilized in this study were taken from high-nutrient (nitrogen-amended) soils and low nutrient-isolates (LNI) taken from low-nutrient (non-amended) soils. Table displays mean characteristic data across three soils samples taken within each plot (n=3). P-values are the result of Welch's two-sample t-tests conducted between high and low nutrient soils with a significance threshold of 0.05 indicated by an asterisk.

**Soil Characteristic**	**Low Nutrient Soils**	**High Nutrient Soils**	**p-value**
pH	6.4	6.0	0.06
Bray P (ppm)	147.3	176.0	0.10
K (ppm)	145.3	177.0	0.06
Total Carbon (% C)	0.7	1.0	0.27
**NO_3-N (ppm)**	**0.7**	**1.5**	**0.04 ***

Table S2: 16S rRNA gene sequence taxonomy assignments for all *Streptomyces* isolates. LNI = Low Nutrient Isolates; HNI = High Nutrient Isolates. Percent identity is based on BLASTN alignment against the NCBI 16S ribosomal RNA database. Reference Accession numbers refer to the top BLASTN hit for each isolate. Query Accession numbers refer to 16S rRNA gene sequences generated in this study and deposited in GenBank.

Isolate	Genus	Species	% Identity	Reference Accession	Query Accession
LNI-1	*Streptomyces*	*S. vinaceus*	100.0%	NR_041131.1	PZ_282427
LNI-2	*Streptomyces*	*S. spororaveus*	100.0%	NR_112469.1	PZ_282428
LNI-3	*Streptomyces*	*S. spororaveus*	99.9%	NR_112469.1	PZ_282429
LNI-4	*Streptomyces*	*S. polychromogenes*	99.8%	NR_041109.1	PZ_282430
LNI-5	*Streptomyces*	*S. olivochromogenes*	99.8%	NR_112483.1	PZ_282431
LNI-6	*Streptomyces*	*S. lannensis*	99.7%	NR_113181.1	PZ_282432
LNI-7	*Streptomyces*	*S. echinatus*	99.0%	NR_112264.1	PZ_282433
LNI-8	*Streptomyces*	*S. echinatus*	99.0%	NR_112264.1	PZ_282434
LNI-9	*Streptomyces*	*S. hokutonensis*	99.8%	NR_134197.1	PZ_282435
LNI-10	*Streptomyces*	*S. rishiriensis*	99.7%	NR_044141.1	PZ_282436
HNI-1	*Streptomyces*	*S. olivochromogenes*	99.8%	NR_112483.1	PZ_282437
HNI-2	*Streptomyces*	*S. mirabilis*	99.7%	NR_041137.1	PZ_282438
HNI-3	*Streptomyces*	*S. mirabilis*	99.7%	NR_041137.1	PZ_282439
HNI-4	*Streptomyces*	*S. mirabilis*	99.9%	NR_041137.1	PZ_282440
HNI-5	*Streptomyces*	*S. sioyaensis*	99.8%	NR_043498.1	PZ_282441
HNI-6	*Streptomyces*	*S. mirabilis*	99.7%	NR_041137.1	PZ_282442
HNI-7	*Streptomyces*	*S. lydicus*	99.9%	NR_112352.1	PZ_282443
HNI-8	*Streptomyces*	*S. olivochromogenes*	99.3%	NR_112483.1	PZ_282444
HNI-9	*Streptomyces*	*S. sioyaensis*	99.8%	NR_043498.1	PZ_282445
HNI-10	*Streptomyces*	*S. spororaveus*	99.9%	NR_112469.1	PZ_282446

Table S3: Minimum inhibitory concentration (MIC μg/ml) of six *Streptomyces*-derived, purified antibiotics across all isolates from low-nutrient (LNI) and high-nutrient (HNI) soils. Subinhibitory concentration of antibiotic (SICA) utilized in experimental work was defined at 10% of isolate MIC value.

Isolate	**Antibiotic Minimum Inhibitory Concentration (μg/ml)**					
	Rifampicin	Tetracycline	Chloramphenicol	Streptomycin	Vancomycin	Streptothricin
LNI-1	0.05	20	20	5	0.12	10
LNI-2	2	20	80	5	0.25	960
LNI-3	2	20	20	0.5	0.5	640
LNI-4	40	160	20	10	0.5	960
LNI-5	10	40	20	0.5	0.5	320
LNI-6	1	2	5	0.5	0.12	2
LNI-7	1	80	80	0.5	2	2
LNI-8	1	80	80	0.5	2	2
LNI-9	20	40	10	0.25	0.5	0.5
LNI-10	1	20	10	0.5	0.25	2
Median	1.5	30	20	0.5	0.5	6.0
Mean	7.80	48.20	34.50	2.33	0.67	289.85
Minimum	0.05	2	5	0.25	0.12	0.5
Maximum	40	160	80	10	2	960
Range	39.95	158.00	75.00	9.75	1.88	959.50

Isolate	**Antibiotic Minimum Inhibitory Concentration (μg/ml)**					
	Rifampicin	Tetracycline	Chloramphenicol	Streptomycin	Vancomycin	Streptothricin
HNI-1	5	80	20	0.5	0.5	2
HNI-2	5	80	10	0.25	5	1
HNI-3	5	80	10	0.5	1	2
HNI-4	2	80	40	0.5	0.5	1
HNI-5	1	40	20	5	0.12	10
HNI-6	5	80	20	0.25	0.5	0.5
HNI-7	20	20	40	5	0.5	320
HNI-8	5	80	20	0.5	1	20
HNI-9	0.5	20	10	5	0.12	10
HNI-10	0.25	2	10	5	0.5	320
Median	5	80	20	0.5	0.5	6.0
Mean	4.88	56.20	20.00	2.25	0.97	68.65
Minimum	0.25	2	10	0.25	0.12	0.5
Maximum	20	80	40	5	5	320
Range	19.75	78.00	30.00	4.75	4.88	319.50

Table S4: Measured carbon sources with carbon type classification defined by the manufacture (Biolog Inc, Hayward CA).

Carbon Class	Carbon Source
Aromatics	2'-deoxy adenosine
Aromatics	Adenosine
Aromatics	inosine
Aromatics	thymidine
Carbohydrate	2,3-butanediol
Carbohydrate	3-methyl-D-glucose
Carbohydrate	a-D-glucose
Carbohydrate	a-D-lactose
Carbohydrate	a-methyl-D-galactoside
Carbohydrate	a-methyl-D-glucoside
Carbohydrate	a-methyl-D-mannoside
Carbohydrate	Amygdalin
Carbohydrate	Arbutin
Carbohydrate	b-methyl-D-galactoside
Carbohydrate	b-methyl-D-glucoside
Carbohydrate	D-arabitol
Carbohydrate	D-cellobiose
Carbohydrate	D-fructose
Carbohydrate	D-galactose
Carbohydrate	D-mannitol
Carbohydrate	D-mannose
Carbohydrate	D-melezitose
Carbohydrate	D-Melibiose
Carbohydrate	D-psicose
Carbohydrate	D-raffinose
Carbohydrate	D-ribose
Carbohydrate	D-sorbitol
Carbohydrate	D-tagatose
Carbohydrate	D-trehalose
Carbohydrate	D-xylose
Carbohydrate	Gentiobiose
Carbohydrate	Glycerol
Carbohydrate	L-arabinose
Carbohydrate	L-fucose
Carbohydrate	l-rhamnose
Carbohydrate	lactulose
Carbohydrate	m-inositol
Carbohydrate	maltose
Carbohydrate	maltotriose
Carbohydrate	N-acetyl-D-glucosamine
Carbohydrate	N-actyl-b-D-mannosamine
Carbohydrate	palatinose
Carbohydrate	Salicin
Carbohydrate	Sedoheptulosan
Carbohydrate	stachyose
Carbohydrate	sucrose
Carbohydrate	turanose
Carbohydrate	xylitol

Carbon Class	Carbon Source
Carboxylic Acid	a-hydroxybutyric acid
Carboxylic Acid	a-ketoglutaric acid
Carboxylic Acid	a-ketovaleric acid
Carboxylic Acid	Acetic acid
Carboxylic Acid	b-hydroxybutyric acid
Carboxylic Acid	D-galacturonic acid
Carboxylic Acid	D-gluconic acid
Carboxylic Acid	D-malic acid
Carboxylic Acid	l-lactic acid
Carboxylic Acid	L-malic acid
Carboxylic Acid	p-hydroxy-phenylacetic acid
Carboxylic Acid	propionic acid
Carboxylic Acid	pyruvic acid
Carboxylic Acid	succinic acid
Carboxylic Acid	y-hydroxybutyric acid
Ester	d-lactic acid methyl ester
Ester	pyruvic acid methyl ester
Ester	succinic acid mono-methyl ester
N-Source	D-alanine
N-Source	glycyl-L-glutamic acid
N-Source	l-alaninamide
N-Source	l-alanine
N-Source	l-alanyl glycine
N-Source	l-asparagine
N-Source	l-glutamic acid
N-Source	L-Pyroglutamic acid
N-Source	L-serine
N-Source	lactamide
N-Source	n-acetyl-L glutamic acid
N-Source	Putrescine
N-Source	succinamic acid
P-Source	a-D-glucose-1-phosphate
P-Source	Adenosine-5'-monophosphate
P-Source	D-fructose-6-phosphate
P-Source	D-glucose-6-phosphate
P-Source	D-L-a-glycerol phosphate
P-Source	Thymidine-5'-monophosphate
P-Source	uridine
P-Source	Uridine-5'monophosphate
Polymer	a-Cyclodextrin
Polymer	b-Cyclodextrin
Polymer	Dextrin
Polymer	Glycogen
Polymer	Inulin
Polymer	Mannan
Polymer	Tween40
Polymer	Tween80

Figure S1: Maximum likelihood phylogenetic tree based on 16S rRNA gene sequences (1,117 bp) of all 20 *Streptomyces* isolates. Bootstrap support values from 1,000 replicates are shown for nodes with ≥80% support. Scale bar represents substitutions per site.

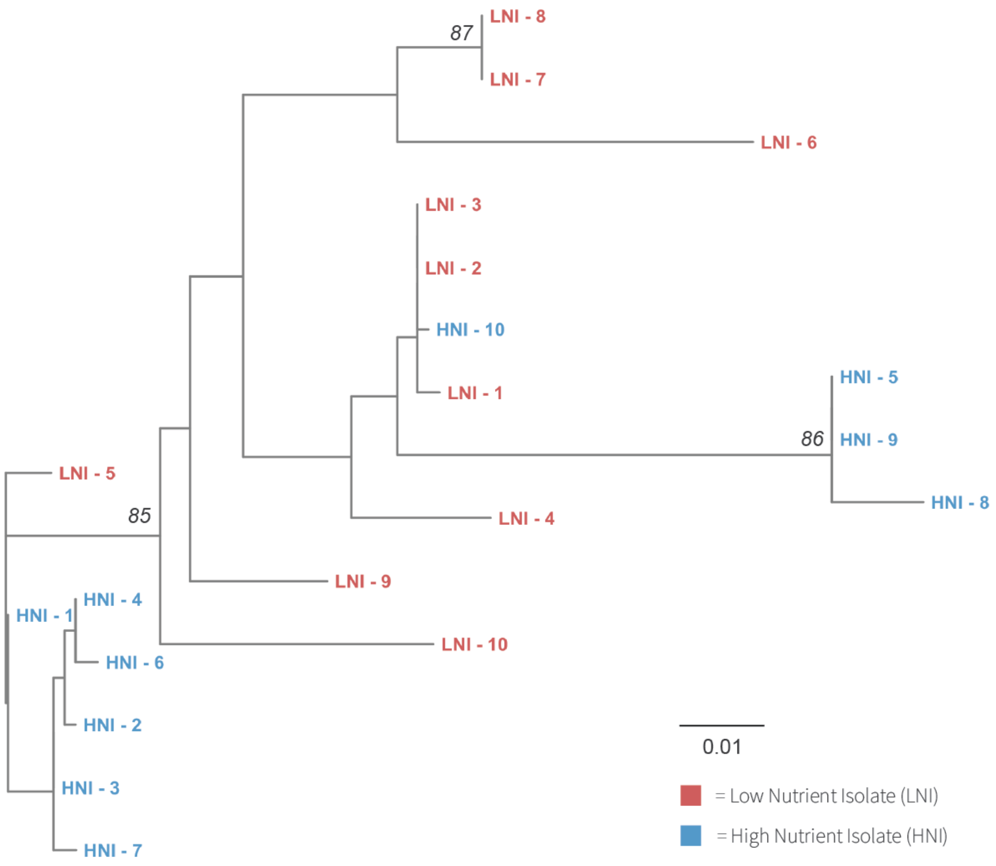

Figure S2: Shifts in isolate growth from control when in the presence of subinhibitory concentration of antibiotic across 95 unique carbon sources among low nutrient isolates (LNI). Each column denotes changes in growth of one isolate. Carbon sources are ordered based on community utilization in the absence of SICA (summed OD_{590} across all isolate growth within each population) with carbon sources with low community utilization (lowest total growth without SICA) are near the bottom, and carbon sources with high community utilization near the top. Heatmap colors denote categorial phenotypes in the presence of SICA with 'induced' defined as no growth in control and growth with SICA, 'increase': greater growth in SICA than control, 'decrease': less growth in SICA than control, and 'suppressed' as growth in control with no growth with SICA. Growth difference values < 1 standard deviation of overall change in growth (< |0.06| from the control) were classified as 'no significant change' and are shown in white. Isolates with low overall growth rate (< 2 total OD_{590}) are not displayed. Accompanies figure 2 in main text.

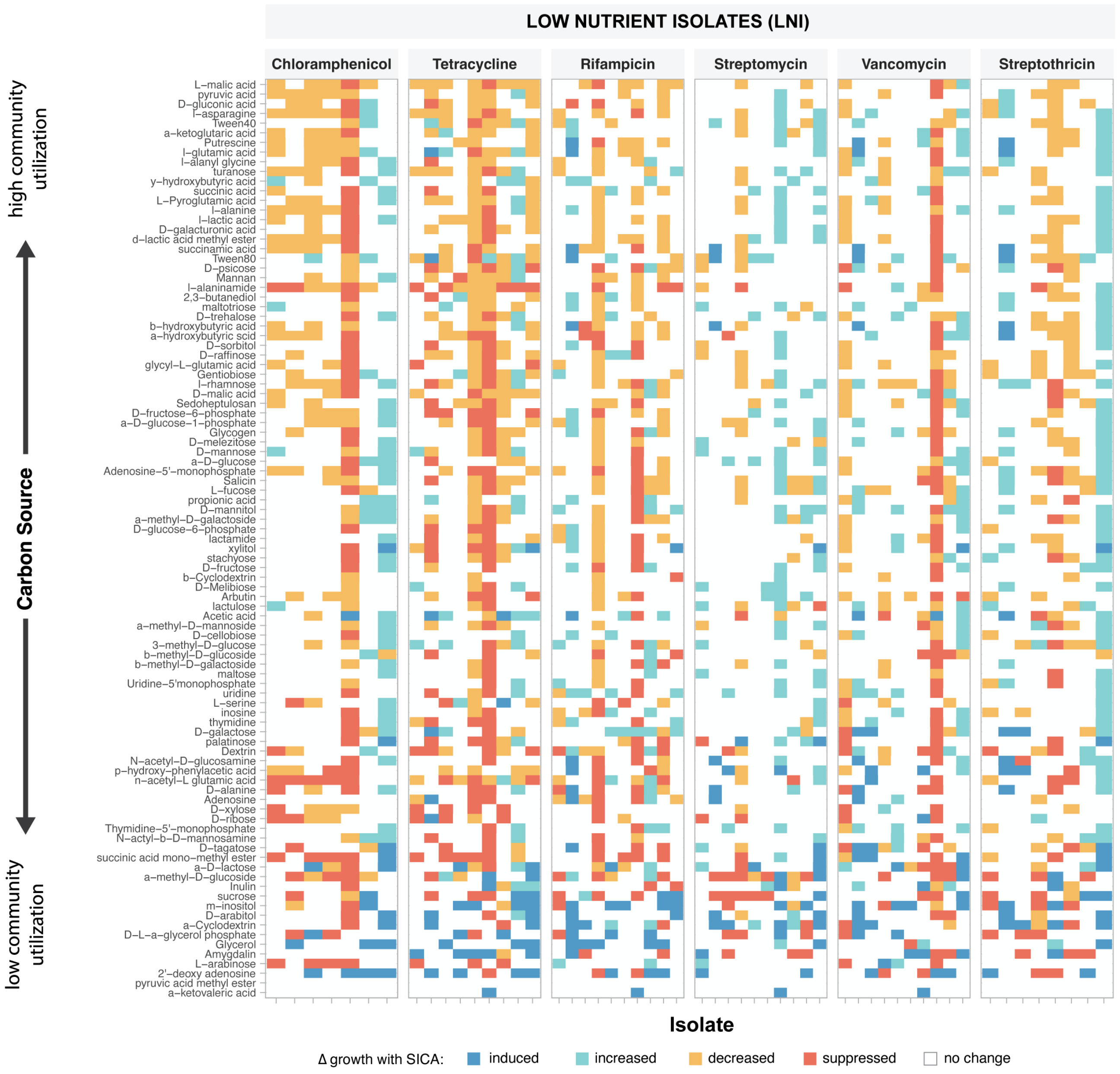

Figure S3: Shifts in isolate growth from control when in the presence of subinhibitory concentration of antibiotic across 95 unique carbon sources among high nutrient isolates (HNI). Each column denotes changes in growth of one isolate. Carbon sources are ordered based on community utilization in the absence of SICA (summed OD_{590} across all isolate growth within each population) with carbon sources with low community utilization (lowest total growth without SICA) are near the bottom, and carbon sources with high community utilization near the top. Heatmap colors denote categorial phenotypes in the presence of SICA with 'induced' defined as no growth in control and growth with SICA, 'increase': greater growth in SICA than control, 'decrease': less growth in SICA than control, and 'suppressed' as growth in control with no growth with SICA. Growth difference values < 1 standard deviation of overall change in growth (< |0.06| from the control) were classified as 'no significant change' and are shown in white. Isolates with low overall growth rate (< 2 total OD_{590}) are not displayed. Accompanies figure 2 in main text.

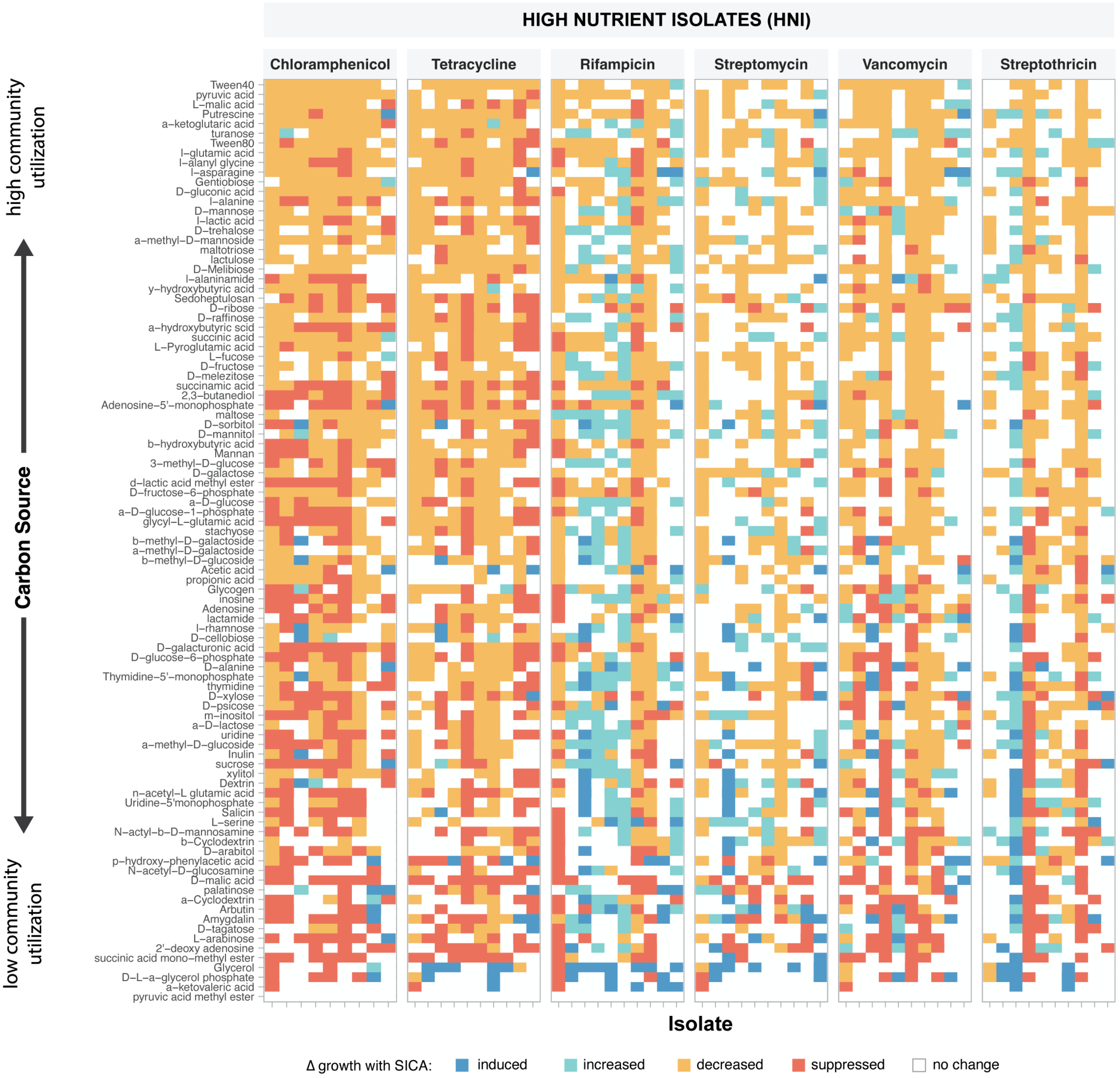

Figure S4: Characterization of changes in competition free growth in the presence of SICA among sympatric low nutrient isolates (LNI) and high nutrient isolates (HNI). Each panel depicts the proportion of escape ratio values across all possible sympatric isolate combinations with >10% change in competition-free growth with SICA. Accompanies figure 3C in the main text.

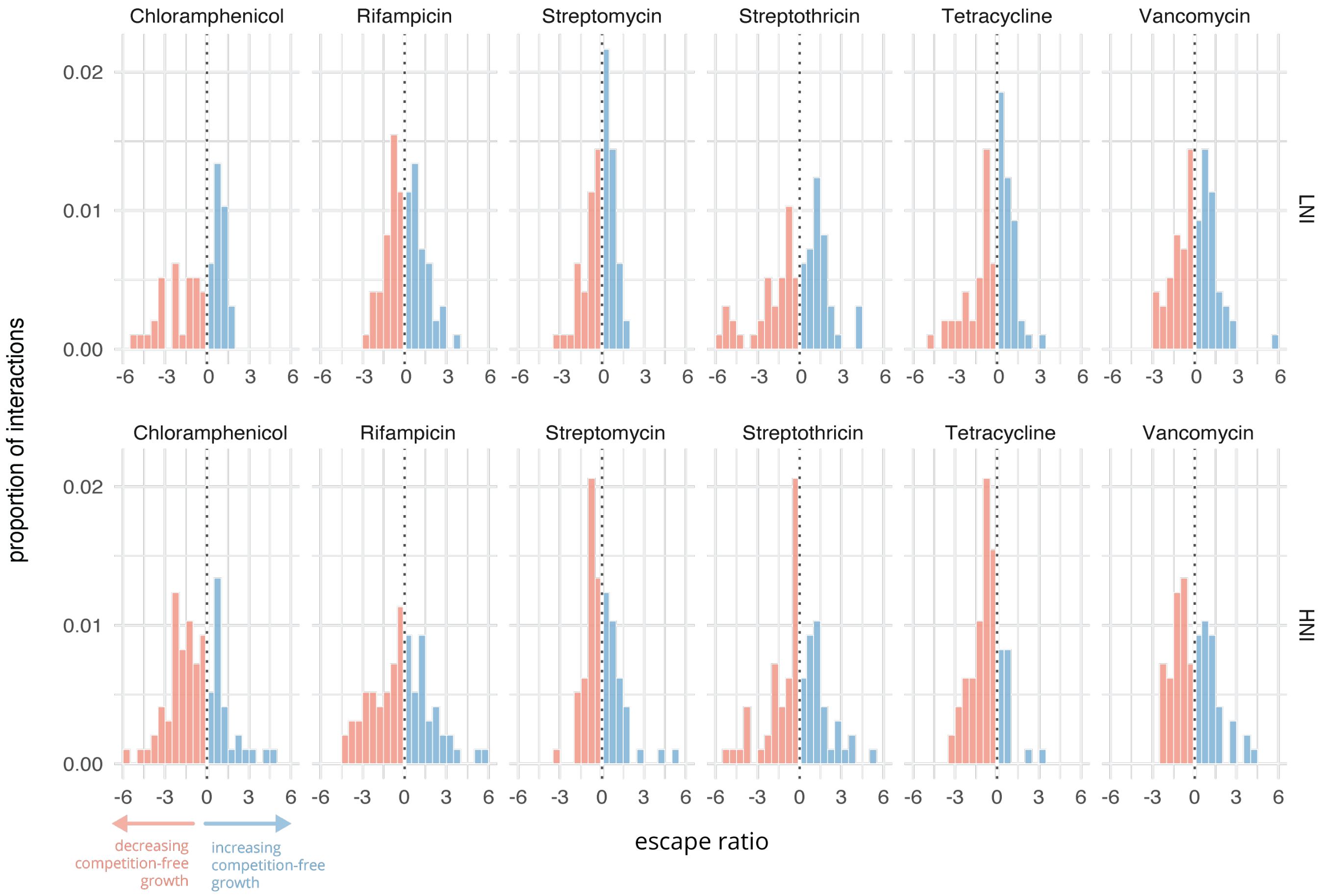

Figure S5: Shifts in isolate growth from control when in the presence of subinhibitory concentration of antibiotic across 95 unique carbon sources among high nutrient isolates (HNI). Each column denotes changes in growth of one isolate. Only isolates with total growth rate > 2 OD_{590} across all carbon sources are displayed. Carbon sources are grouped by carbon classification defined in table S2. Heat map colors denote difference in isolate growth in the presence vs absence of SICA. Only growth difference values >1 standard deviation from overall population mean (>|0.06 OD_{590}|) were classified as significant change and colored on the heatmap.

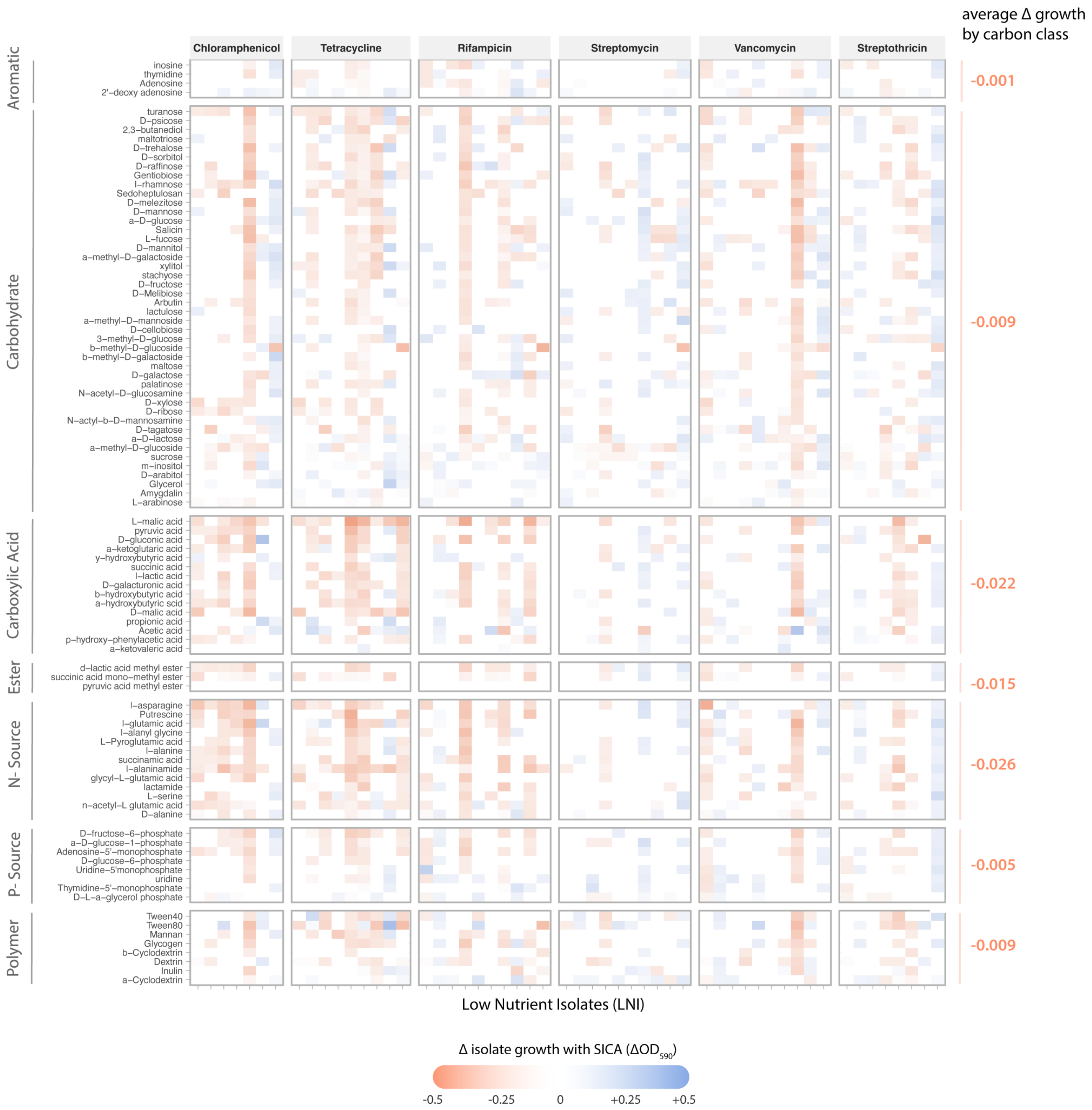

Figure S6: Shifts in isolate growth from control when in the presence of subinhibitory concentration of antibiotic across 95 unique carbon sources among low nutrient isolates (LNI). Each column denotes changes in growth of one isolate. Only isolates with total growth rate > 2 OD_{590} across all carbon sources are displayed. Carbon sources are grouped by carbon classification defined in table S2. Heat map colors denote difference in isolate growth in the presence vs absence of SICA. Only growth difference values >1 standard deviation from overall population mean (>|0.06 OD_{590}|) were classified as significant change and colored on the heatmap.

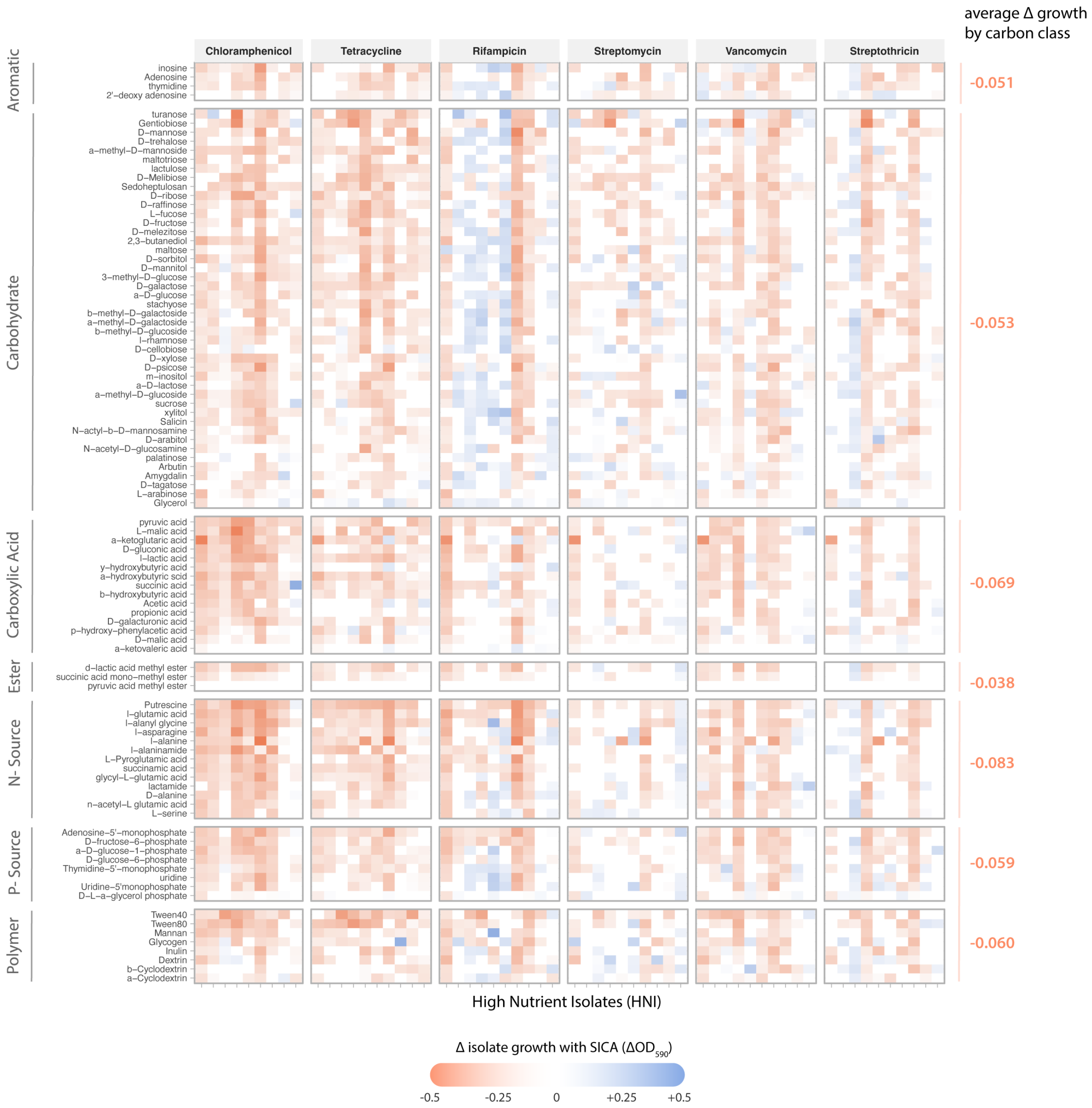

Figure S7: Mean change in isolate growth in the presence of subinhibitory concentrations of antibiotics (SICA) across all measured carbon classes. Bars display mean change in isolate growth (OD_{590}) averaged across 6 antibiotics, with error bars + 1SE from mean. Blue denotes high-nutrient isolates (HNI), red denotes low-nutrient isolates (LNI). The breakdown of individual carbon sources within each class can be found in table S4 and figures S7 and S8.

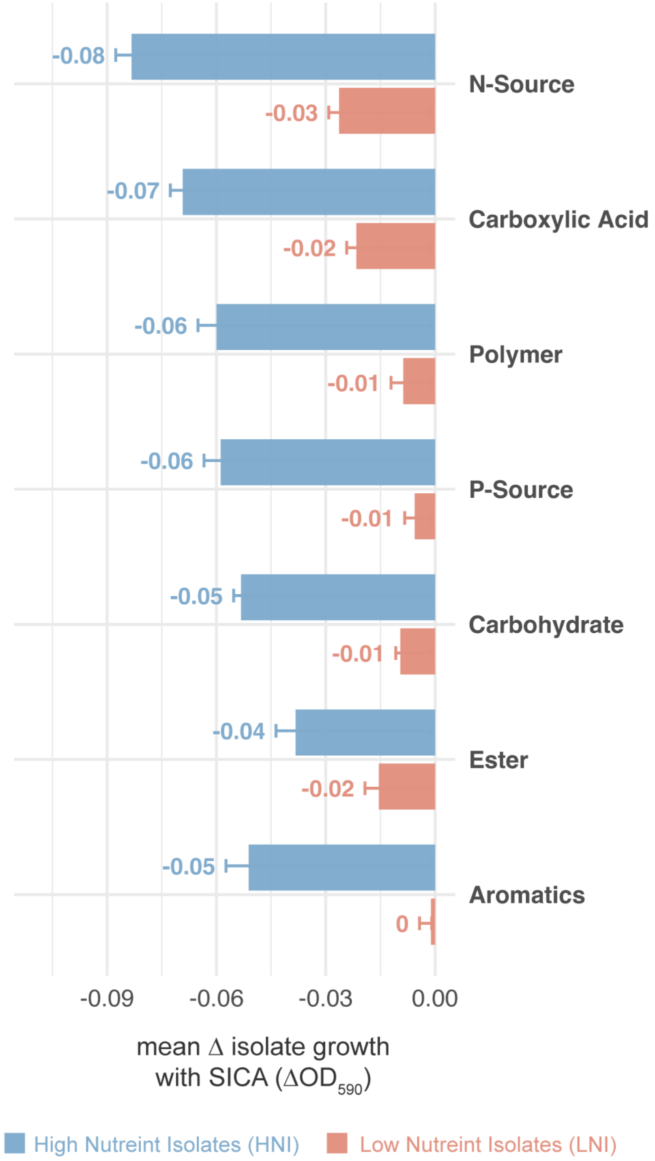

Figure S8: Mean niche width for low (LNI) and high nutrient isolates (HNI) in both the absence of subinhibitory concentrations of antibiotics (SICA, control, n = 10/bar) and the presence of SICA (with SICA, n=60/bar). Bars depict +/-1 standard deviation from mean, p value denotes significance from Welch 2-sample t-test.

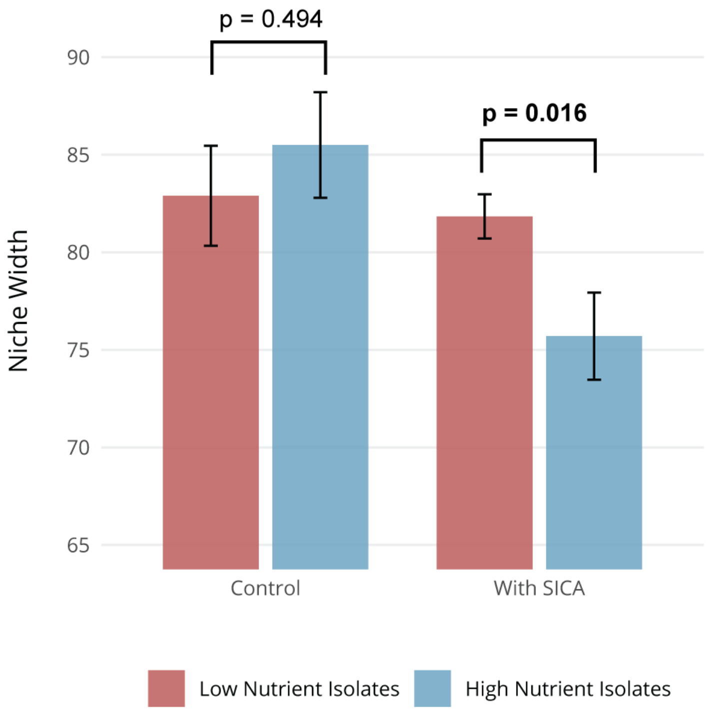

Supplement: KuhsSICA_supplement_final_wrag121 [file kuhssica_supplement_final_wrag121.pdf]
